# HLA‐B27 association of autoimmune encephalitis induced by PD‐L1 inhibitor

**DOI:** 10.1002/acn3.51213

**Published:** 2020-10-08

**Authors:** Hyeyeon Chang, Yong‐Won Shin, Bhumsuk Keam, Miso Kim, Seock‐Ah Im, Soon‐Tae Lee

**Affiliations:** ^1^ Department of Neurology Seoul National University Hospital Seoul Republic of Korea; ^2^ Department of Neurology Konyang University Hospital Deajeon Republic of Korea; ^3^ Center for Hospital Medicine, Seoul National University Hospital Seoul Republic of Korea; ^4^ Department of Neurosurgery Seoul National University Hospital Seoul Republic of Korea; ^5^ Department of Internal Medicine Seoul National University Hospital Cancer Research Institute Seoul National University College of Medicine Seoul Republic of Korea

## Abstract

**Objective:**

While immune checkpoint inhibitors are increasingly used for various cancers, unpredictable immune‐related adverse events (irAEs) such as autoimmune encephalitis is life‐threatening. Here, we report an association between human leukocyte antigen (HLA) and atezolizumab‐induced encephalitis.

**Methods:**

From an institutional prospective cohort for encephalitis, we identified patients with autoimmune encephalitis after the use of atezolizumab, a PD‐L1 (programmed death‐ligand 1) inhibitor, from August 2016 to September 2019 and analyzed their HLA genotypes.

**Results:**

A total of 290 patients received atezolizumab, and seven patients developed autoimmune encephalitis, and five of whom were enrolled for the analysis. The patients presented altered mentality, seizures, or myelitis. Three patients had the HLA‐B*27:05 genotype in common (60%), which is significantly frequent given its low frequency in the general population (2.5%). After Bonferroni correction, HLA‐B*27:05 was significantly associated with autoimmune encephalitis by atezolizumab (corrected *P* < 0.001, odds ratio 59, 95% CI = 9.0 ~ 386.9).

**Interpretation:**

Here we found that three in five patients with autoimmune encephalitis associated with atezolizumab had the rare HLA‐B*27:05 genotype. Further systematic analyses in larger cohorts are necessary to investigate the value of HLA screening to prevent the life‐threatening adverse events.

## Introduction

Immune checkpoint inhibitors (ICPIs) are increasingly used as an effective immune‐based cancer therapy option in many types of solid and hematologic cancers.^1^ These drugs inhibit immune checkpoints on the effector T cell or tumor side, such as CTLA‐4, PD‐1, and PD‐L1. However, by activating effector T cells and recognizing autoantigens,^2^ ICPIs can cause immune‐related adverse events (irAEs), such as inflammation in the gastrointestinal tract, endocrine glands, skin, lung, liver, and nervous system.^3^ Guillain–Barre syndrome, myasthenia gravis, and aseptic meningitis are known irAEs in the nervous system,^4^, ^5^, ^6^, ^7^ and central nervous system (CNS) involvement in irAEs sometimes causes encephalitis or myelitis, resulting in permanent disability or fatality.^8^, ^9^, ^10^, ^11^

The exact mechanism by which some patients develop encephalitis, a serious CNS‐irAE, and the patients who are at a high risk for this complication remainunknown. Atezolizumab is a PD‐L1 inhibitor approved for non‐small‐cell lung cancer,^12^, ^13^ urethral carcinoma,^14^ and advanced triple‐negative breast cancer.^15^, ^16^ Although the incidence of irAEs caused by atezolizumab is believed to be lower than that caused by other ICPIs,^3^, ^17^ several serious encephalitis cases have been reported after the use of atezolizumab.^8^, ^9^, ^18^, ^19^ However, no known risk factors for irAEs were identified. Recently, we observed five consecutive patients with encephalitis caused by atezolizumab and found that they have a unique human leukocyte antigen (HLA) genotype. Here, we show evidence that HLA is likely a risk factor for encephalitis caused by atezolizumab.

## Methods

### Patients

We generated a prospective cohort of patients with encephalitis at Seoul National University Hospital and analyzed patients with autoimmune encephalitis after the use of a PD‐L1 inhibitor (atezolizumab) from August 2016 to September 2019. Two expert neurologists in autoimmune menigoencephalitis (S‐T.L. and H.C.) determined the relationship between atezolizumab and encephalitis based on the subacute development of CNS‐irAEs and the diagnostic criteria for autoimmune encephalitis.^20^ The association between ICI use and the encephalitis was evaluated by the Naranjo algorithm.^21^ All patients underwent brain MRI, tests to measure blood urea nitrogen, creatinine, electrolytes, cobalamin, methylmalonic acid, homocysteine, folate, lactate, ammonia, and creatinine phosphokinase, porphyria screening, urine analysis, liver function tests and a cerebrospinal fluid (CSF) study. In addition, CSF bacterial and fungal cultures and CSF polymerase chain reaction for viruses (herpes simplex viruses 1 and 2, varicella‐zoster virus, Epstein–Barr virus, cytomegalovirus, human herpesviruses 6 and 8, enterovirus, respiratory virus, and JC virus) were performed. Immunotherapy to control irAEs was administered following the appropriate guidelines^22^ and was adjusted according to the patients’ AE presentations. Outcomes were measured by the modified Rankin scale (mRS) 1 and 3 months after symptom onset. This study was approved by the Seoul National University Hospital Institutional Review Board (IRB approval number: 1705‐130‐856) and complied with the principles of the Declaration of Helsinki.

### HLA genotyping

We extracted genomic DNA from the patients' blood and performed HLA genotyping. The genotype DNA sequencing of the HLA‐A, HLA‐B, HLA‐C, HLA‐DRB1, and HLA‐DQB1 genes of each subject was analyzed using direct DNA sequence analysis according to an established protocol (Biowithus, Seoul, Korea). The subjects were analyzed at the 4‐digit allele level. Reported HLA frequencies in the Korean population were used as a control group.^23^

### Statistical analysis

For statistical analysis, we used a control group of HLA genotypes from 485 Korean general populations. For each HLA type, odds ratios with 95% confidence intervals were calculated. The Fisher extract test was used to correlate patients with specific HLA types and CNS‐irAEs. Calibration for multiple tests was performed by multiplying the P‐value by the number of alleles detected at each HLA locus: 44 for the HLA‐B alleles and 22 for the HLA‐C alleles (Bonferroni method). Statistical analysis was performed using SPSS version 18 (SPSS Inc., Chicago, IL, USA), and a two‐sided *P*‐value < 0.05 was considered significant.

### Data Availability Statement

Individual participant data that underline the results reported in this article will be available from the corresponding author on request, in anonymized form.

## Results

### Patient characteristics

**FIGURE 1:**
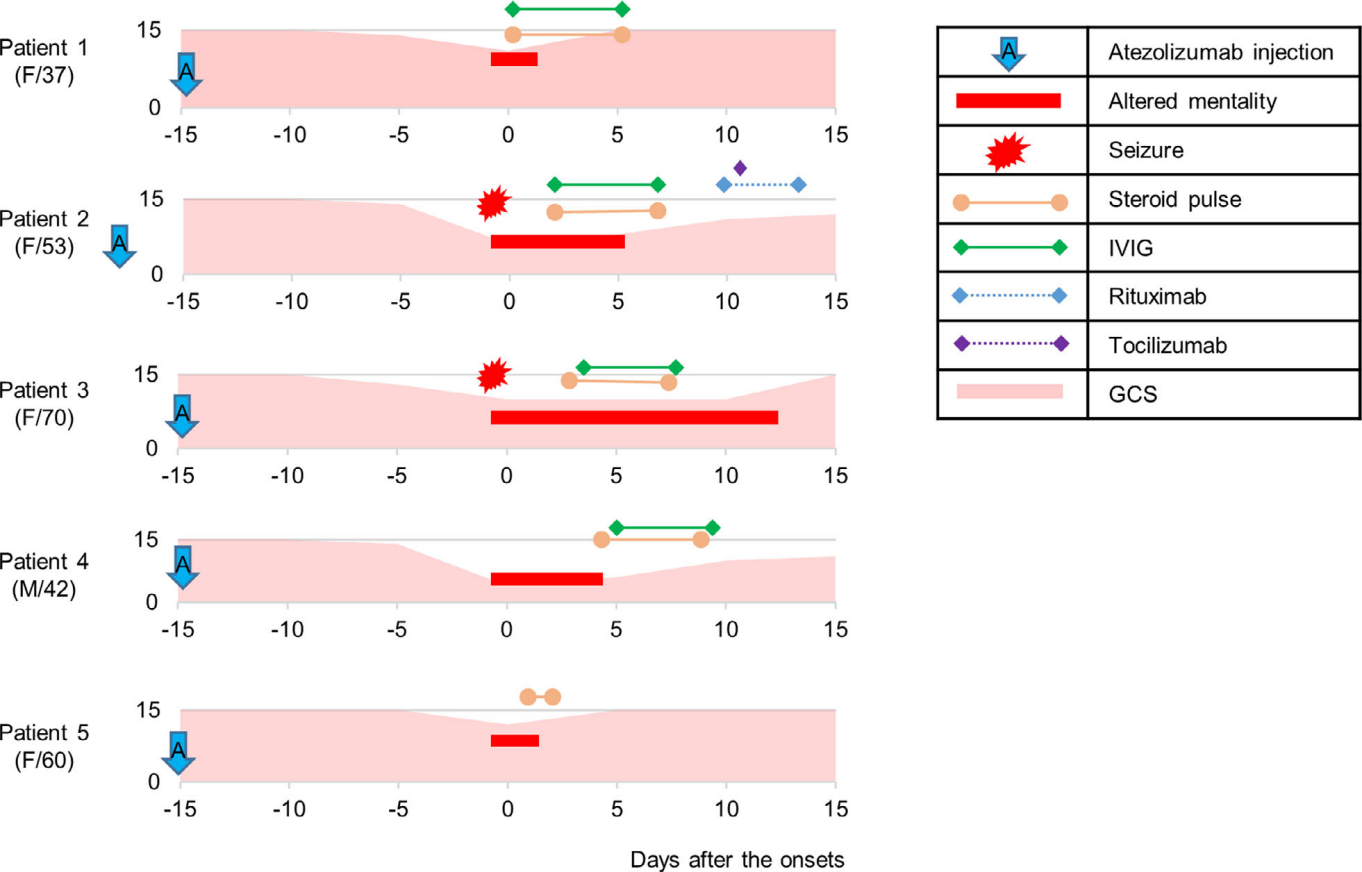
Clinical courses and treatment outcomes of the patients. GCS = Glasgow Coma Scale, IVIG = intravenous immunoglobulin, F = female, M = male.

**FIGURE 2:**
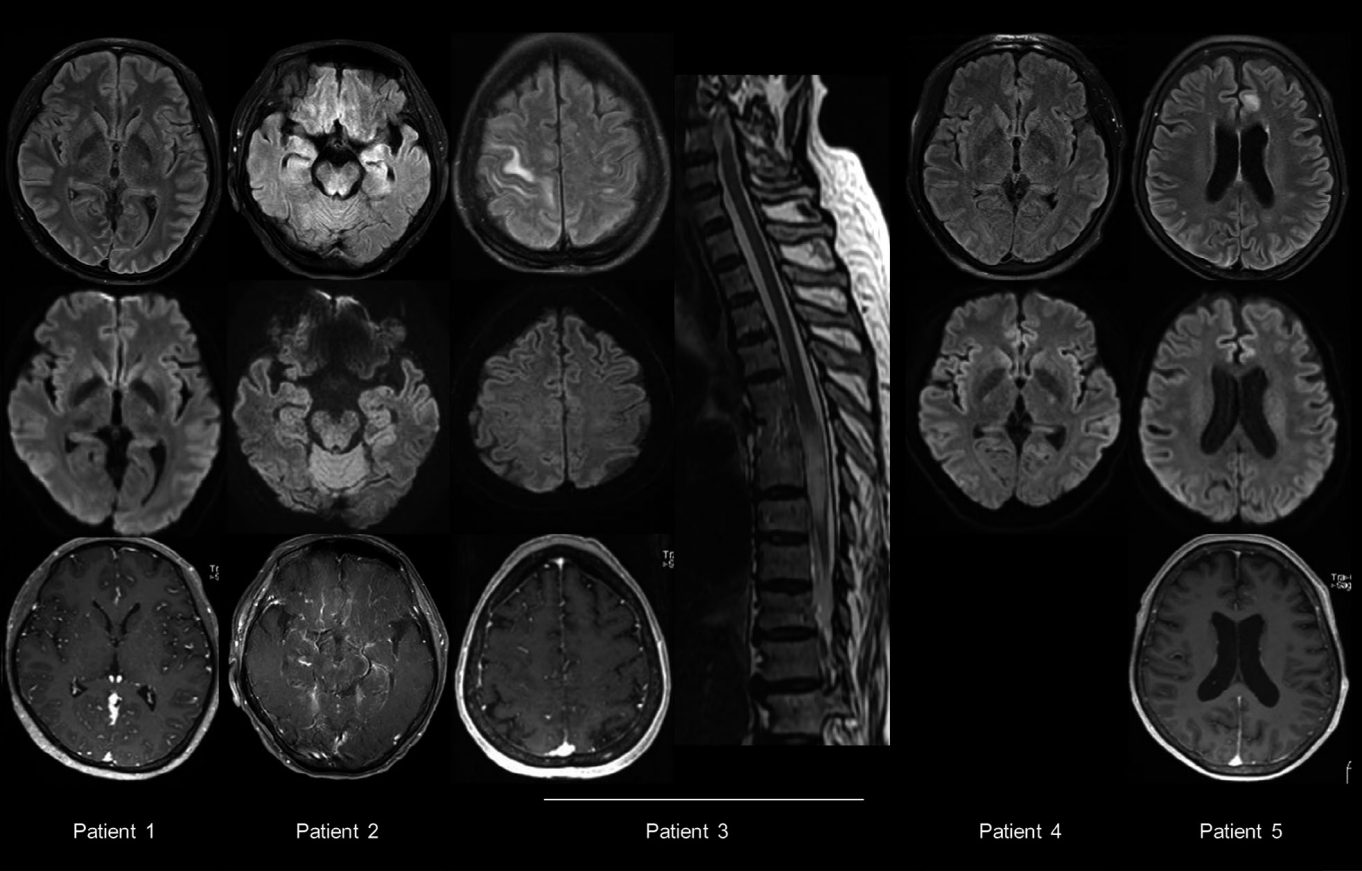
Lesions on MRI. Upper row: Diffusion‐weighted images. Middle row: Fluid‐attenuated Inversion Recovery Sequence. Lower row: Gadolinium‐enhanced T1‐weighted images, spine MRI of Patient 3: Ill‐defined T2 hyperintense intramedullary abnormality of the spinal cord at the T6 ~ T9 level.

During the study period, a total of 290 patients received atezolizumab, seven of whom developed subacute encephalitis after atezolizumab administration. Two patients could not be enrolled in the current HLA study because DNA samples were not available (due to technical failure of DNA extraction for one patient, and the other patient did not provide consent for the study). Accordingly, a total of five patients were enrolled in the analysis, and their clinical characteristics are summarized in Table 1 and Fig 1. All patients had score 7 (problable) in the Naranjo adverse drug reaction probability scale.^21^ The initial symptoms, such as fever, fatigue, and headache, developed at days 5 ~ 15 after atezolizumab administration and progressed to decreased consciousness at days 15 ~ 16. Two patients had seizures. All the patients also had systemic irAEs. MRI showed diffuse leptomeningeal enhancement indicating meningitis and T2 high signals in the neocortex (patients 2, 3, and 5) (Fig. 2). CSF showed pleocytosis and increased protein levels. Four patients (Patient 1 ~ 4) were checked for paraneoplastic antibodies against amphiphysin, CRMP5, Ma2, Ri, Yo, Hu, Recoverin, Sox‐1, Titin, Zic4, and glutamic‐acid decarboxylase‐65, which were all negative. Patient 2 was tested for antibodies against NMDA‐R, leucine‐rich‐glioma‐inactivated 1 (LGI‐1), CASPR2, α‐amino‐3‐hydroxy‐5‐methyl‐4‐isoxazolepropionic‐acid‐receptor (AMPAR), dipeptidyl‐peptidase–like protein 6 (DPPX), and γ‐aminobutyric‐acid‐receptor‐B (GABAB), which were all negative.

**Table 1:** Clinical presentations, treatment outcomes, and HLA association of the patients.

	Patient 1	Patient 2	Patient 3	Patient 4	Patient 5
Sex/Age	F/37	F/53	F/70	M/42	F/60
Type of cancer	Breast cancer	Bladder cancer	Bladder cancer	Bladder cancer	Breast cancer
Previous chemotherapy	None	Gemcitabine, cisplatin, paclitaxel, pemetrexed	Bacillus Calmette–Guerin (BCG), gemcitabine, cisplatin	Gemcitabine, cisplatin	Doxobubicin, cyclophosphamide, palbociclib,
Combined chemotherapy	Cobimetinib	None	None	Cobimetinib	Fulvestrant, ipatasertib
Clinical phenotype	Encephalitis (fever, altered mentality)	Encephalitis (fever, seizure, cranial nerve palsy), axonal Guillain–Barre syndrome (limb weakness, facial palsy)	Encephalitis (fever, seizure, altered mentality), Guillain–Barre syndrome‐motor dominant (limb weakness, facial palsy), myelitis (incontinence, saddle anesthesia)	Encephalitis (fever, altered mentality)	Encephalitis (fever, altered mentality)
Systemic irAE	Skin rash, hepatitis, pneumonitis	Hepatitis, pneumonia	Skin rash	Diarrhea	Skin rash, hepatitis, mucositis, eosinophilia
Seizure	(‐)	(+)	(+)	(‐)	(‐)
EEG abnormality	Not performed	Diffuse slowing	Diffuse slowing	Diffuse slowing	Diffuse slowing
From atezolizumab to mental change (day)	15	18	15	15	16
CSF findings	‐ Pleocytosis (WBC 54/μl, poly 51%, lympho 40%, other 7%), high protein (111 mg/dL), glucose (76mg/dL)	18cmH_2_O Pleocytosis (WBC 222/μl, poly 38%, lympho 15%, other 47%), high protein (1000 mg/dL), glucose (81mg/dL)	18cmH_2_O Pleocytosis (WBC 30/μl, poly 20%, lympho 20%, other 60%), high protein (358 mg/dL), glucose (79mg/dL)	27cmH_2_O Pleocytosis (WBC 65/μl, poly 0%, lympho 11%, other 89%), high protein (133 mg/dL), glucose (78mg/dL)	13cmH_2_O Pleocytosis (WBC 135/μl, poly 5%, lympho 50%, other 45%), high protein (377 mg/dL), glucose (86mg/dL)
MRI findings	Diffuse leptomeningeal enhancement	T2 high signals in limbic and brainstem areas with leptomeningeal enhancement	T2 high signals in white matter (right> left) and T6 ~ T9 spinal cord	No significant abnormality	T2 high signals in the left medial frontal gyrus with leptomeningeal enhancement
Immunotherapy against irAE	Steroid, immunoglobulin	Steroid, immunoglobulin, rituximab, tocilizumab	Steroid, immunoglobulin, rituximab	Steroid, immunoglobulin	Steroid
Time required to recover consciousness (days)	2	6	4	5	2
Initial mRS	5	5	5	5	5
mRS at 1 month	2	5	5	2	2
mRS at 3 months	2	5	5	2	2
HLA‐B*27:05	Negative	Positive	Positive	Positive	Negative
HLA‐C1*01:02	Negative	Positive	Positive	Positive	Positive

After treatment with immunotherapy, mainly with steroids (methylprednisolone 1 g daily for 5 days) and intravenous immunoglobulin (IVIG, 2 g/kg over 5 days), or with rituximab and tocilizumab in some serious patients (patients 2 and 3), consciousness was recovered in 2 ~ 6 days. However, disability measured by mRS scores remained unchanged.

### HLA genotypes of the patients with CNS‐irAEs

After analyzing the full HLA genotypes of the five patients and comparing them to those in the normal Korean population, we found that two HLAs were increased in the CNS‐irAE patients. The first genotype was HLA‐B*27:05, which was identified in the three patients (60%) with the most severe clinical phenotype (patients 2, 3, and 4). In the Korean population, HLA‐B27 is present in only 3.1% of individuals,^24^ and HLA‐B*27:05 is present in only 2.5% of individuals.^25^ After Bonferroni correction, HLA‐B*27:05 was significantly associated with CNS‐irAEs (corrected *P* < 0.001, odds ratio 59, 95% CI = 9.0 ~ 386.9) (Table 2). HLA‐C1*01:02 was also found in 4 of 5 patients (80%, patients 2, 3, 4, and 5). HLA‐C1*01:02 is present in 18% of the Korean population. The association between HLA‐C1*01:02 and CNS‐irAEs was not significant after Bonferroni correction (corrected *P* = 0.088, odds ratio 18.8, 95% CI = 2.0–170.5). The Table S1 provides the detailed HLA genotypes.

**Table 2:** Frequency differences in selected alleles in CNS‐irAE patients versus healthy controls.

HLA allele frequency	CNS‐irAE patient‐ no. (%)	Healthy controls‐ no. (%)	OR(95% CI)	*P*	Pc
HLA‐B*27:05	3/5 (66%)	12/485 (2.5%)	59.1 (9.0–386.9)	<0.001	<0.001
HLA‐C1*01:02	4/5 (80%)	89/485 (18%)	18.8 (2.0–170.5)	0.004	0.088

* HLA data are from five CNS‐irAE patients and 485 healthy controls. Pc: Pc values are the results of modifications using Bonferroni's method for multiple comparisons. For correction, P‐values were multiplied by the number of alleles detected for each HLA locus. HLA = human leukocyte antigen.

### Clinical phenotypes of patients with HLA‐B*27:05

The three patients (patients 2, 3, and 4) with HLA‐B*27:05 developed encephalitis with altered mentality, two of whom were considered severe and showed decreased mentality or myelitis (mRS = 4). Patient 2 had meningitis, limbic and brainstem encephalitis, cranial nerve palsies, and Guillain–Barre syndrome. This patient received ICU care because of seizures, and MRI showed multiple T2 signal changes in the brainstem. Steroids and IVIG were not sufficient for treatment, and rituximab and tocilizumab were added. The patient remained disabled and died after 7 months due to cancer progression. Patient 3 had encephalitis, myelitis, and Guillain–Barre syndrome. Myelitis involved the T6 ~ T9 levels, and the patient remained paraplegic even after steroid, IVIG, and rituximab treatment. Patient 4 had encephalitis and improved 5 days after steroid and IVIG treatment. None of the three patients with HLA‐B*27:05 had experienced any symptoms of ankylosing spondylitis.

## Discussion

Here, we found that three in five patients with CNS‐irAEs caused by atezolizumab have the HLA‐B*27:05 genotype. The population frequency of HLA‐B*27:05 is only 2.5% in Koreans and 3.7% in the United States. and European Caucasians.^26^ The expected number of HLA‐B*27:05 in 290 atezolizumab‐treated Korean patients is about seven (7.25). We found that three had CNS‐irAE. In addition, the patients with HLA‐B*27:05 had a severe phenotype of the CNS‐irAE. Because CNS‐irAEs are life‐threatening when develops, the finding has significant clinical implications and warrant in‐depth multicenter investigations to determine whether serious CNS‐irAEs can be prevented if HLA‐B*27:05 is included in the screening test before the use of ICPIs.

Encephalitis induced by irAEs causes seizures, altered mentality, and memory loss.^3^ Encephalitis induced by irAE can be diagnosed by the clinical phenotype, MRI, and exclusion of infectious encephalitis. Sometimes, autoantibodies such as anti‐NMDAR and anti‐Hu can be detected.^9^, ^10^ Treatment of encephalitis induced by irAEs includes steroids, IVIG and more advanced immunomodulators such as rituximab and tocilizumab.^22^, ^27^ While most patients respond to steroid and guidelines suggest to use steroid first,^20^ patients with Guillain–Barre syndrome need IVIG and those with severe refractory diseases could be controlled by combination immunotherapy using biological immunosuppressants,^27^ as we did in our patient #2 and #3. Although CNS‐irAEs are possible with all ICPIs, including CTLA‐4, PD‐1, and PD‐L1 inhibitors,^3^, ^17^ this study analyzed a PD‐L1 inhibitor because autoimmune encephalitis cases by the other drugs were rare in our institution. All five patients in this study showed the typical clinical presentation of autoimmune encephalitis, which could be explained most likely by the use of atezolizumab.

Originally, the HLA‐B*27:05 genotype was associated with ankylosing spondylitis and other systemic inflammatory diseases, such as psoriasis, inflammatory bowel disease, and reactive arthritis.^28^ While the exact mechanism by which HLA‐B*27 induces inflammatory disease is not exactly known, HLA‐B27 may be an antigen pocket for molecular mimicry^29^ and may be recognized by CD4 T cells to initiate a proinflammatory response through an unfolded protein response.^30^ The self‐peptide displayed by HLA‐B27 may be the target of autoreactive CD8 T cells.^31^, ^32^ In addition, HLA‐B*27:05 enhances the expression of the costimulatory molecule B27,^28^, ^33^ which is necessary for T‐cell activation by ICPIs. The mechanism by which HLA‐B27 (especially the HLA‐B*27:05 genotype) contributes to CNS‐irAEs requires further study. In addition, whether the HLA‐B*27:05 genotype influences the antitumor efficacy of ICPIs is another issue of interest.

If patients treated with atezolizumab have the HLA‐B*27:05 genotype, the risk of CNS‐irAEs can to be discussed. In similar, HLA‐B*15:02 and HLA‐A*31:01 increase the risk of Stevens–Johnson syndrome or toxic epidermal necrolysis in patients treated with carbamazepine or oxcarbazepine.^34^, ^35^, ^36^ Thus the guideline by the Clinical Pharmacogenetics Implementation Consortium (CPIC) recommends not to use carbamazepine if these HLAs are detected.^37^ Similarly, HLA‐DRB1*11:01 might be associated with pruritus due to ICPI use.^38^ In this study, Ali et al. analyzed 102 patients who received ICI treatment with CTLA‐4 and PD‐1, and the most common single side effect was pruritis. They found significant associations between HLA‐DRB1*11:01 and pruritus and between HLA‐DRB1*03:01 and colitis.

However, we have no data regarding whether HLA‐B*27:05 is associated with other systemic irAEs caused by atezolizumab or whether HLA‐B*27:05 increases the risk of irAEs induced by other ICPIs, such as CTLA‐4 or PD‐1 inhibitors. In addition, because patients with HLA‐B27 or ankylosing spondylitis have an slightly increased risk of hematologic malignancy or systemic cancer,^39^, ^40^ it is also necessary to analyze the prevalence of HLA‐B27 in the control cancer patients. Until then, our data suggest that HLA‐B*27:05 might be a risk factor gene for CNS‐irAEs induced by atezolizumab. Because the current case number in our study is limited, prospective systematic analysis of HLA genotypes in patients receiving ICPIs is necessary to clarify the association and to prevent disabling adverse effects by autoimmune encephalitis.

## Authors’ Contributions

H.C. and S.‐T.L. involved in the study concept, design, and experiments. All authors involved in data acquisition and analysis, drafting of the manuscript, and figures. All authors participated in the revision of the manuscript and approved the final version.

## Conflicts of Interest

Patent pending for the HLA analysis to predict CNS‐irAE (H.C., and S.‐T.L.). Seock‐Ah Im received a research grant from AstraZeneca, Pfizer and Roche outside of the current work and have advisory role for AstraZeneca, Amgen, Eisai, Hanmi Corp. Lilly, Novartis, Pfizer, and Roche outside of the current work. Soon‐Tae Lee have an advisory role for Ono Pharmaceuticals and received research grants from GC Pharma outside of the current work.

## Supporting information


**Table S1.** HLA genotypes of the patients.
