# Supplementary material for: HLA‐B27 association of autoimmune encephalitis induced by PD‐L1 inhibitor
Source: Ann Clin Transl Neurol. 2020 Oct 8;7(11):2243–50. doi: 10.1002/acn3.51213 (PMC7664281; doi:10.1002/acn3.51213)
Supplement: Supplementary file 1 — Table S1. HLA genotypes of the patients. [file ACN3-7-2243-s001.docx]

**Supporting Information**

**Table S1. HLA genotypes of the patients.**

| HLA alleles | A | A | B | B | C | C | DRB1 | DRB1 | DQB1 | DQB1 |
| --- | --- | --- | --- | --- | --- | --- | --- | --- | --- | --- |
| Patient 1 | 03:02:01G | 24:02:01G | 08:01:01G | 40:06:01G | 07:02:01G | 08:01:01G | 03:01:01G | 15:01:01G | 02:01:01:01G | 06:02:01:01G |
| Patient 2 | 02:06:01G | 33:03:01G | **27:05:02G** | 44:03:01:01G | **01:02:01G** | 14:03 | 01:01:01G | 13:02:01G | 05:01:01:01G | 06:04:01:01G |
| Patient 3 | 02:06:01G | 30:01:01G | **27:05:02G** | 40:06:01G | **01:02:01G** | 08:01:01G | 04:03:01G | 12:01:01G | 03:02:01:01G | 03:02:01:01G |
| Patient 4 | 02:01:01G | 26:02:01 | **27:05:02G** | 15:01:01G | **01:02:01G** | 03:03:01G | 01:01:01G | 14:06:01 | 03:01:01:01G | 05:01:01:01G |
| Patient 5 | 02:01G | 33:03G | 15:18G | 44:03G | **01:02G** | 14:03G | 08:02G | 13:02G | 04:02G | 06:04G |
